# Meta-analysis of identified genomic regions and candidate genes underlying salinity tolerance in rice (*Oryza sativa* L.)

**DOI:** 10.1038/s41598-024-54764-9

**Published:** 2024-03-08

**Authors:** Pratik Satasiya, Sanyam Patel, Ritesh Patel, Om Prakash Raigar, Kaushal Modha, Vipul Parekh, Haimil Joshi, Vipul Patel, Ankit Chaudhary, Deepak Sharma, Maulik Prajapati

**Affiliations:** 1 Department of Genetics and Plant Breeding, N. M. College of Agriculture, Navsari Agricultural University, Navsari, Gujarat India; 2 School of Agricultural Biotechnology, Punjab Agricultural University, Ludhiana, Punjab India; 3 Kishorbhai Institute of Agriculture Sciences and Research Centre, Uka Tarsadia University, Bardoli, Gujarat India; 4 Department of Biotechnology, College of Forestry, Navsari Agricultural University, Navsari, Gujarat India; 5 Coastal Soil Salinity Research Station Danti-Umbharat, Navsari Agricultural University, Navsari, Gujarat India; 6 Regional Rice Research Station, Vyara, Navsari Agricultural University, Navsari, Gujarat India

**Keywords:** Genome-wide meta-QTL, QTL, Salinity stress, Confidence interval, Salinity tolerance, Consensus map, Candidate genes, Biochemistry, Biotechnology, Molecular biology, Plant sciences

## Abstract

Rice output has grown globally, yet abiotic factors are still a key cause for worry. Salinity stress seems to have the more impact on crop production out of all abiotic stresses. Currently one of the most significant challenges in paddy breeding for salinity tolerance with the help of QTLs, is to determine the QTLs having the best chance of improving salinity tolerance with the least amount of background noise from the tolerant parent. Minimizing the size of the QTL confidence interval (CI) is essential in order to primarily include the genes responsible for salinity stress tolerance. By considering that, a genome-wide meta-QTL analysis on 768 QTLs from 35 rice populations published from 2001 to 2022 was conducted to identify consensus regions and the candidate genes underlying those regions responsible for the salinity tolerance, as it reduces the confidence interval (CI) to many folds from the initial QTL studies. In the present investigation, a total of 65 MQTLs were extracted with an average CI reduced from 17.35 to 1.66 cM including the smallest of 0.01 cM. Identification of the MQTLs for individual traits and then classifying the target traits into correlated morphological, physiological and biochemical aspects, resulted in more efficient interpretation of the salinity tolerance, identifying the candidate genes and to understand the salinity tolerance mechanism as a whole. The results of this study have a huge potential to improve the rice genotypes for salinity tolerance with the help of MAS and MABC.

## Introduction

Rice is an important contributor to global food security as it sustains half of the world's population, yet abiotic factors continue to threaten its production. Among the abiotic factors, salinity stress is one of the most brutal factors governed by many quantitative traits responsible for phenotypic and physiological phenomena^1^. The seedling stage is the most vulnerable stage, with even minor stress of 5–6 EC causing stress symptoms. The plant exhibits significant symptoms such as reduced growth and development, osmotic destabilization, ROS accumulation and ionic imbalance when exposed to salinity. These are the key responsible factors behind the reduced growth of leaves and roots at seedling stages as it affects root length, diameter, ionic (Na^+^, K^+^ and Cl^-^) and hormonal concentrations etc.^2,3^. Apart from the seedling stage, the reproductive stage is the second most important stage where the salinity stress affects anther development, pollen production, spikelet sterility, flag leaf ionic concentrations etc. which ultimately translated into a reduction in grain yield per plant^4^.

Much research has been conducted to discover the genomic regions (QTLs) responsible for salinity tolerance, which is the most essential feature to improve the genotypes for better plant growth and development under saline environments^5–8^. Despite the difficulties of phenotyping salinity tolerance at the reproductive stage, considerable efforts were undertaken to discover the major QTLs governing these traits^9–12^. Currently one of the most significant challenges in using QTLs to improve crop resistance against salinity stress, is determining which QTLs have the best chance of improving salinity tolerance with the least amount of background noise from the tolerant parent. To do so, minimizing the QTL confidence interval (CI) is desirable to include the responsible genes for salinity stress tolerance, it does not guarantee finding only those genes in the interval.^13^.

One of the important approaches is to use meta-QTLs (MQTLs) that identifies the stable and consensus regions to increase the efficiency of genomics assisted breeding (GAB) as it provides unique advantages over the other approaches of mapping QTLs^14^. To date, many attempts have been made to identify the MQTL regions for many crop species^15–19^. As the MQTLs were derived from the different genetic backgrounds and included phenotyping across the environments to identify the consensus genomic regions responsible for salinity tolerance, the results of the meta-QTL analysis are highly reliable and can be used successfully in any GAB program^20–22^. The MQTL analysis reduces the confidence interval (CI) to many folds from the initial QTL studies unrevealing the possibilities to identify the candidate genes underlying the region that is responsible for the salinity tolerance^23^.

In the present study, a comprehensive genome-wide meta-QTL analysis was performed using the data retrieved from the QTL mapping studies, GBS study and the GWAS study of the past 22 years to identify the MQTLs responsible for salinity tolerance at the seedling and reproductive stage of rice. The results of the MQTLs could be applicable to any of the GAB programmes to improve the genotypes for tolerance to salinity and stability of the transferred region across the environments and genetic backgrounds. The functional annotation was done for the screen the MQTL regions to identify the candidate genes responsible for salinity tolerance in rice. The results of the pan-genome analysis indicated the same set of genes responsible for the stress responses and its mechanisms to overcome it^24^. So, the syntenic regions in wheat and barley were identified for mining the orthologous genes responsible for salinity tolerance. The identified orthologues can be potentially used for functional analysis and characterization for tolerance to salinity across the species.

## Materials and methods

### Initial studies of QTL mapping for MQTL analysis

**Figure 1:**
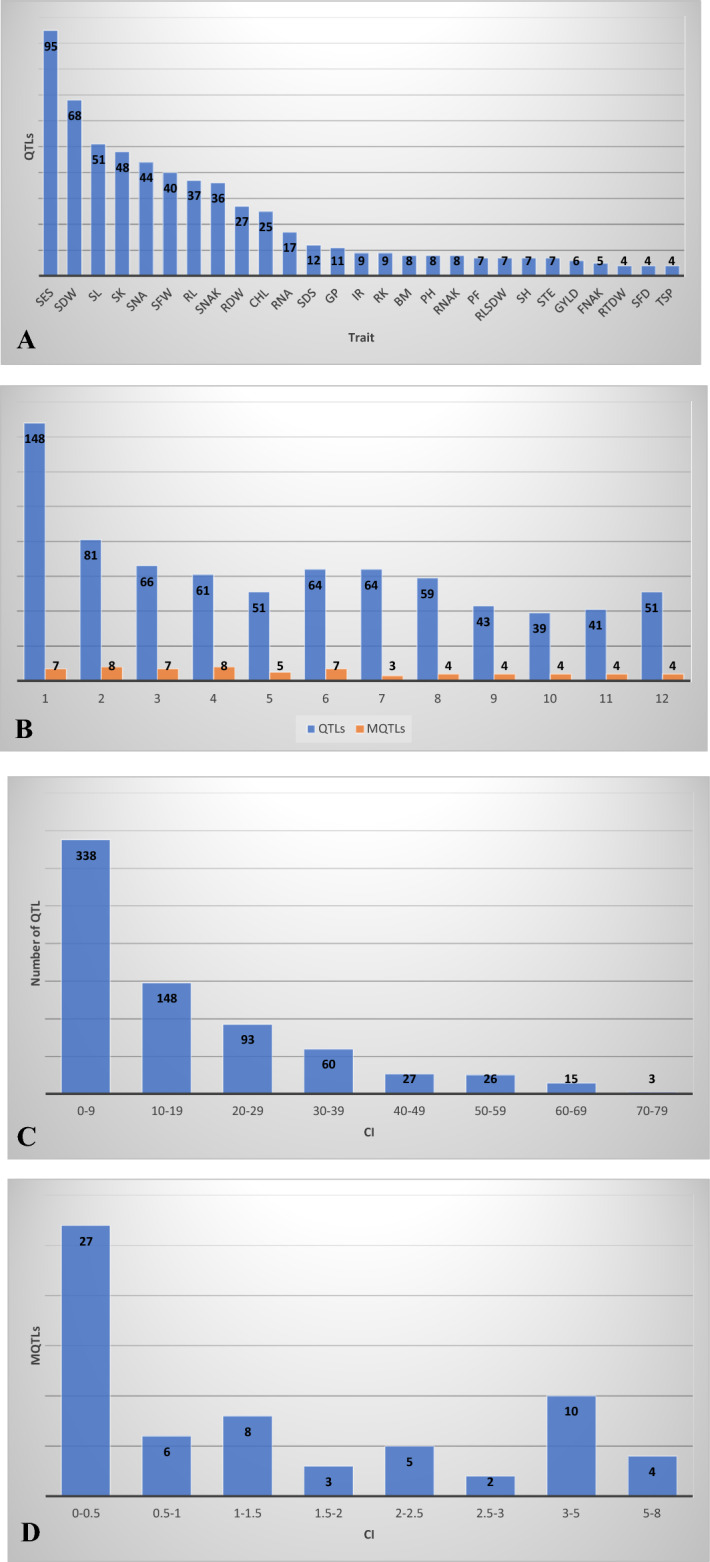
(**A**) QTLs retrieved from 35 independent mapping populations and used for MQTL analysis for salinity tolerance at seedling and reproductive stages (SES: Salt evaluation score, SDW: Shoot dry weight, SL: Shoot length, SK: Shoot K^+^ concentration, SNA: Shoot Na^+^ concentration, SFW: Shoot fresh weight, RL: Root length, SNAK: Shoot Na^+^/K^+^ concentration, RDW: Root dry weight, CHL: Chlorophyll content, RNA: Root Na^+^ concentration, SDS: Survival days, GP: Germination per cent, IR: Imbibition rate, RK: Root K^+^ concentration, BM: Biomass, PH: Plant height, RNAK: Root Na^+^/K^+^ concentration, PF: Pollen fertility, RLSDW: Relative shoot dry weight, SH: Shoot height, STE: Spikelet sterility, FNAK: Flag leaf Na^+^/K^+^ concentration, RTDW: Relative total dry weight, SFD: Shoot fresh/dry weight, TSP: Total spikelet). (**B**) Chromosome wise QTLs and MQTLs distribution on twelve chromosomes of rice. (**C**) Frequency distribution of the QTLs based on confidence intervals for seedling and reproductive stage salinity tolerance. (**D**) Frequency distribution of the MQTLs based on confidence intervals for seedling and reproductive stage salinity tolerance.

An exhaustive preview of the QTL mapping studies was carried out for the seedling stage and reproductive stage salinity tolerance in rice from 2001 to 2021 (Fig. 1A). Thirty-four studies having thirty-five independent mapping populations with all the information on mapping population, its size, molecular markers, genetic map, LOD score and the phenotypic variance explained (R^2^) were selected for the MQTL analysis. The studies with the missing information were excluded from the analysis. The mapping populations used in the studies were generated from the cross combinations of 51 susceptible and tolerant parents and the Genome Wide Association Mapping Study (GWAS) included 179 landraces evaluated under saline conditions. The size of the mapping population ranges from 62 to 281 and the molecular markers used for generating the genetic map were ranges from 29 to 56,897. The studies included various types of genotyping platforms *i.e.*, marker-based linkage mapping, Array-based, GWAS and GBS which utilized various molecular markers viz., RFLP, AFLP, InDel, ESTs, SSRs and SNPs. A total of 768 QTLs were extracted for 71 different traits correlated with salinity tolerance at seedling and reproductive stages viz., 95 for salt evaluation score (SEC), 68 for root dry weight (RDW), 51 for shoot length (SL), 50 for shoot K^+^ concentration (SKC), 40 for shoot Na^+^ concentration (SNC) etc. The traits related to salinity tolerance were regrouped into morphological attributes and ionic concentrations (Na^+^, K^+^ and Na^+^/K^+^ ratio) for which combined and separate analysis was conducted. Detailed information regarding the parents of the mapping populations, population type, population size, marker used, marker type and the traits under study are represented in Tables 1 and 2.

**Table 1:** Summary of the studies of QTL mapping used for MQTL analysis for salinity tolerance in rice.

Sr. no.	Parents	Population type	Population size	No. of markers	Marker type	Trait	References
1	Nona Bokra/Koshihikari	F_2:3_	133	161	RFLP	SDS, SNA, NAU, SK, RNA, RNAU, RK, RKU	Lin et al. (2004)^63^
2	Tesanai 2/CB	RILs	96	74	RFLP	SDS, SDW, RDW, SNA, SK, SNAK	Masood et al. (2004)^64^
3	Jiucaiqing/IR36	F_2_	250	62	SSR	SES, RNAK, SDW	Ming-zhe et al. (2005)^65^
4	Co39/Moroberekan	RILs	120	29	SSR	SFW, SFD, SFW, SDW, SFD	Haq et al. (2008)^66^
5	CSR27/MI-48	F_2_	200	275	SSR, EST	SES, NAU, KU, NAKL, NAKS, CLU	Ammar et al. (2009)^67^
6	Ilpumbyeo/Moroberekan	BC_3_F_5_	117	125	SSR	RDW, RFW, RLA, RST	Kim et al. (2009)^68^
7	CSR 27/MI 48	RILs	216	162	SNP, SSR	NKU, KU, NAKS, NAU, NCLV, SES, NNAU	Pandit et al. (2010)^7^
8	Tiqing (TQ) / Tarome-M	BC_2_F_5_	62	114	SSR	SK, RK, RNA, SNAK, RNAK	Ahmadi et al. (2011)^46^
9	BRRI dhan40/NSIC Rc106	F_2_	93	262	EST, SSR	SES	Islam et al. (2011)^69^
10	Shaheen Basmati/Pokkali	F_2:3_	190	108	SSR	SL, SFW, SDW, SNA, SK, SNAK, RNA, RK, RNAK, SES	Javed et al. (2011)^70^
11	Teqing/wild rice (O rufipogon)	ILs	87	95	SSR	SES, RLRDW, RTDW	Tian et al. (2011)^71^
12	IR26/Jiucaiqing	RILs	150	135	SSR	SH, DSW, SH, DRW	Wang et al. (2011)^72^
13	IR26/Jiucaiqing	RILs	150	135	SSR	IR, GP	Wang et al. (2012)^73^
14	Gharib (T)/Sepidroud (S)	F_2_	148	236	AFLP, SSR	RFW, SFW, RDW, BM, SES, RL, SNA, SK, SNAK, CHL	Ghomi et al. (2013)^74^
15	Sadri/FL478	F_2_	232	123	SSR	DTF, PH, PN, DM, NFS, STE, TSP, GYLD, PF, TGW	Mohammadi et al. (2013)^75^
16	Cheriviruppu/PB 1	F_2_	218	131	SSR	PH, TN, PN, BM, PF, FNA, FNAK	Hossain et al. (2015)^9^
17	IR4630/IR15324	RILs	118	119	AFLP	NAU, SK, SNAK, KU, SNA, DM	Koyama et al. (2001)^76^
18	Ce258/ZGX1	ILS	200	179	SSR	SES, SDS, SNA, SK	Qiu et al. (2015)^77^
19	Bengal/Pokkali	ILS	276	107	SSR	SES, SL, RL, SDW	De Leon et al. (2016)^78^
	Bengal/Pokkali	RILs	187	1650	SNP	SNA, SK, SNAK, SES, CHL, SL, RL, SDW	
20	At354 (T)/ Bg352 (S)	RILs	281	1135	SNP	SNAK, SNA, SK, SES, SL, RL, SFW, RFW, SDW, RDW	Gimhani et al. (2016)^79^
21	IR36/Pokkali	F_2_	113	111	SSR	GLWR, TGW, HI, UGP, DMS, STR	Khan et al. (2016)^80^
22	IR29/Hasawi	RILs	142	194	SNP	SES, RL, SL, SFW, RFW, SDW, RDW	Bizimana et al. (2017)^81^
23	Bengal/Pokkali	ILs	72	6797	SNP	SK, SNAK, SES, CHL, SL, SDW	De Leon et al. (2017)^82^
24	Jupiter/Nona Bokra	ILs	138	126	SSR	SES, SNA, SK, SNAK, CHL, SL, RL, SDW	Puram et al. (2017)^83^
25	IR29/Hasawi	RILs	145	194	SNP	SES, SL, RL, SFW, SDW	Rahman et al. (2017)^84^
26	Cheniere/Nona Bokra	ILs	112	116	SSR	SES, SK, SNAK, CHL, SL, RL, SDW, RLSNA, RLSL, RLRL, RLSDW	Puram et al. (2018)^85^
27	NSIC Rc222/BRRI dhan 47	F_2_	92	87	SNP	PH, TN, NFS, TSP, SFP, GYLD	Mondal et al. (2019)^10^
28	BRRI dhan29/Capsule	F_2_	94	105	SSR, InDel	SES, SDS, SL, SFW, SNA, SK, SNAK, CHL	Rahman et al. (2019)^86^
29	AC41585/IR 64	BC_3_F_5_	180	109	SSR	DEG, SES, STE	Chattopadhyay et al. (2021)^12^
30	IR29/Pokkali	RILs	148	56,897	SNP	SES, SNA, SK, SKNA, RNA, RK, RKNA, SDW, SFW, SL	Chen et al., (2020)^87^
31	Kolajoha/Ranjit	RILs	68	1248	SNP	SNA, RNA, NAK, SES, RL, SL, RFW, RSW, PDW	Mazumder et al. (2020)^88^
32	Dongnong425/Changbai10	BC_2_F_2_	190	137	SSR	SES, SNA, SK, RNA, RK	Zhang et al. (2020b)^8^
33	Landraces	LRs	179	21,623	SNP	SES, SNA, SNAK, CHL, SK	Le et al. (2021)^30^
34	PS5/CSR10	F_2_	140	100	HvSSR	SES, PH, PL, PT, BM, GYLD, CHL, PRO, RNA, RK, RNAK, SK, SNAK, FNA, FNAK, SSI, STI	Pundir et al. (2021)^11^

**Table 2:** The traits for which The QTLs compiled.

Sr. no.	Trait	No. of QTLs	Trait name	Sr. no.	Trait	No. of QTLs	Trait name
Morphological traits							
1	SES	95	Salt evaluation score	11	BM	8	Biomass
2	SDW	68	Shoot dry weight	12	PH	8	Plant height
3	SL	51	Shoot length	13	RTDW	4	Relative total dry weight
4	SFW	40	Shoot fresh weight	14	SFD	4	Shoot fresh/dry weight
5	RL	37	Root length	15	TSP	4	Total spikelet
6	RDW	27	Root dry weight	16	PF	7	Pollen fertility
7	CHL	25	Chlorophyll content	17	RLSDW	7	Relative shoot dry weight
8	SDS	12	Survival days	18	SH	7	Shoot height
9	GP	11	Germination per cent	19	STE	7	Spikelet sterility
10	IR	9	Imbibition rate	20	GYLD	6	Grain yield
Traits related to ionic stress							
1	SES	95	Salt evaluation score	5	RNA	17	Root Na^+^ concentration
2	SK	48	Shoot K^+^ concentration	6	RK	9	Root K^+^ concentration
3	SNA	44	Shoot Na^+^ concentration	7	RNAK	8	Root Na^+^/K^+^ concentration
4	SNAK	36	Shoot Na^+^/K^+^ concentration	8	FNAK	5	Flag leaf Na^+^/K^+^ concentration

The successful integration of the Genome-Wide Association Study (GWAS) into the meta-analysis was achieved through a meticulous process involving the conversion of base pair distances into centimorgans (cM), facilitating linkage map preparation. Subsequently, the p-values, serving as indicators of the significance of marker-trait associations, underwent a transformation into logarithm of odds (LOD) scores using the following equation^25^.

$${\text{LOD}} = - \log_{10} (P)$$

Additionally, to ascertain the representative position of each distinct QTL, the mean position was computed based on the flanking markers, offering an accurate characterization of their genomic location within the meta-analysis framework. Through this comprehensive approach, all the essential information necessary for the meta-analysis was successfully amassed.

The confidence interval (95%) for each of the QTL was calculated as the difference between the left and right positions of the QTLs. Wherever the positions were not given it was calculated by the formula ${\text{CI}}={\text{X}}/({\text{N}}\times {{\text{R}}}^{2})$, where the X is 530 for BC and F_2_, 287 for DH lines and 163 for RILs and ILs, N is the size of the population and R^2^ is the phenotypic variance explained^26^.

### Preparation of consensus map and projection of QTLs

The consensus map was prepared using the genetic map information from all the 35 maps and the reference maps retrieved from the supplementary tables 17 and 18 of the IRGSP, 2005 (https://archive.gramene.org), Orjuela et al. 2010 and Cornell SSR 2001^27^. The reference maps are having marker density of 8740 markers with an average map of 1527.22 cM and the average length of each chromosome is 127.27 cM. The consensus genetic map was prepared with the help of the LPmerge (Ver. 1.7) software that uses linear programming with ensuring that the order of the markers is preserved as in the linkage maps^28^. All the 768 QTLs were successfully projected on the consensus map as per the information available viz., CIs, LOD score R^2^, peak positions and flanking positions with the help of the software BioMercator V4.2.3^22^.

### QTL meta-analysis

**Figure 2:**
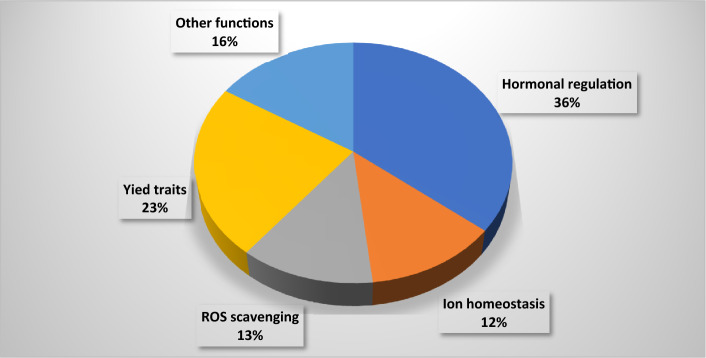
Candidate genes identified based on their potential role in salinity tolerance in rice.

Veyrieras et al. (2007) two-step algorithm was used to carry out the meta-analysis of the QTLs projected on the consensus map using the software BioMercator V4.2.3^29^. By using the information of the Akaike Information Criterion (AIC), corrected Akaike Information Criterion (AICc and AIC3), Bayesian Information Criterion (BIC) and Average Weight of Evidence (AWE) criteria, we have selected the consistent value to choose the best model for identifying the number of MQTL or true QTLs. Statistical procedures and their algorithms were thoroughly described by Sosnowski et al. (2012)^22^ (Fig. 2).

### Identification and functional analysis of candidate genes for salinity tolerance

**Figure 3:**
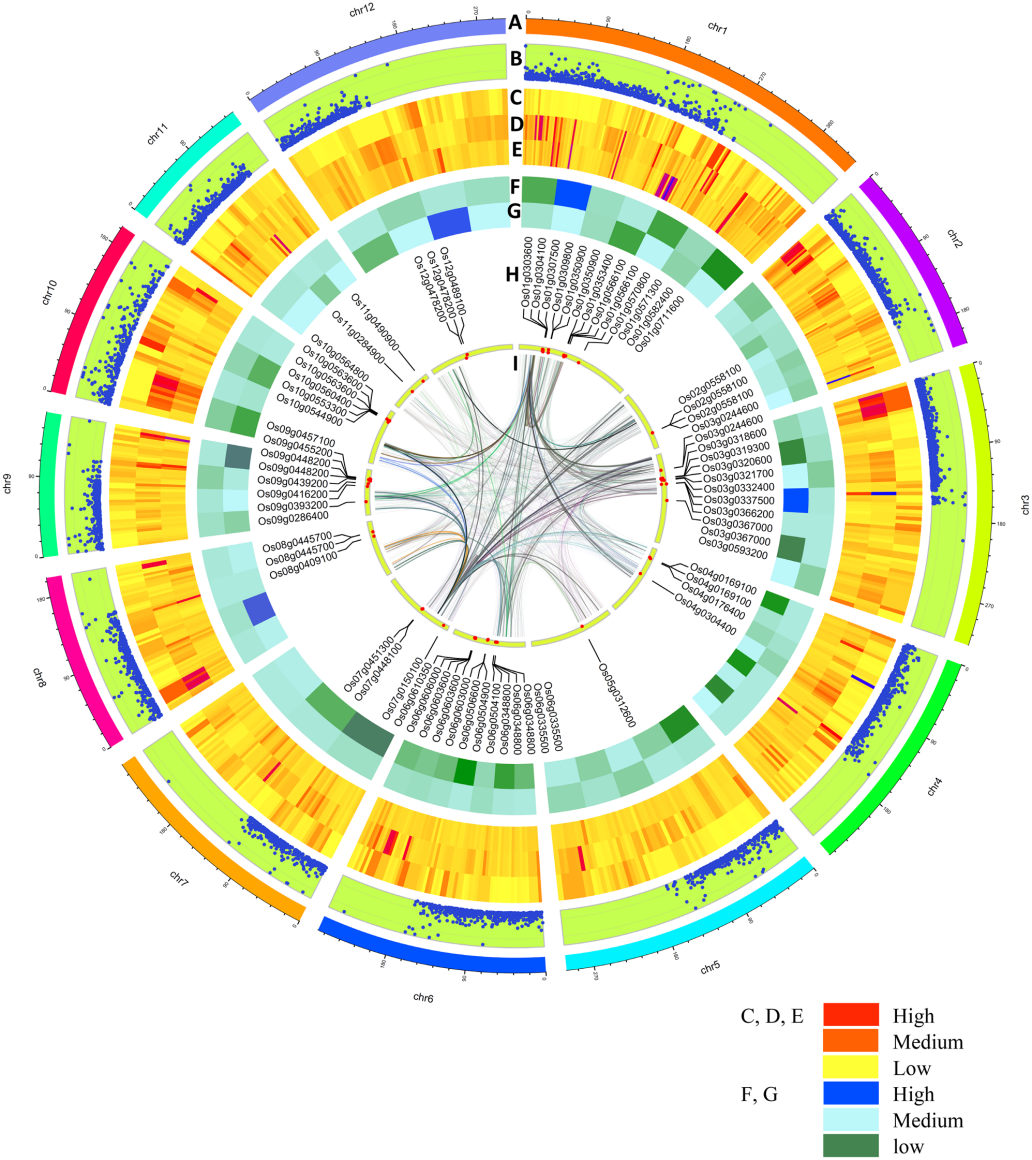
Visualization of circos plot after MQTL analysis (**A**) Chromosome number (**B**) Marker density (**C**) LOD of the initial QTLs (**D**) R2 of initial QTLs (**E**) Confidence interval of initial QTLs (**F**) Number of candidate QTLs (**G**) Confidence interval of MQTLs (**H**) Genes identified in MQTL region (**I**) Homologous regions of the identified genes.

The MQTLs selected for the functional analysis for candidate gene identification based on the criteria of greater than 5 candidate QTLs with an average LOD score of 3 and phenotypic variance of more than 8 per cent. The region of the MQTL for functional annotation is calculated by adding and subtracting the CI on either side of the identified MQTL and the nearest marker to that region is selected. The marker position is retrieved from the GWAS study in base pairs and used as the start and endpoint of the MQTL region^30^. The MQTL region which does not have the flanking SNPs were physically located using the Rice Annotation Project Database (RAPDB) (http://rapdb.dna.affrc.go.jp), or the sequence of forward and reverse primer is subjected to nucleotide blast in NCBI (https://blast.ncbi.nlm.nih.gov/Blast.cgi?PROGRAM=blastn) to detect the sequence in the Nipponbare reference genome. The candidate genes underlying that region was investigated on the genome (IRGSP-1.0) using “Biomart” of the Ensemble plants database and the Gramene (https://archive.gramene.org/qtl/). The retrieved GO terms are enriched by removing the redundant terms and summarized with the help of Revigo (http://revigo.irb.hr/). After identification of candidate genes in MQTL region, FunRiceGenens (https://funricegenes.github.io/) and Ensamble Plants (https://plants.ensembl.org/index.html) databases were searched to functionally annotate these genes. All the aforementioned information about the consensus map, QTL density, MQTLs identified for salinity tolerance and the candidate genes were shown in the circus plot (Fig. 3).

### Identification of orthologous genes in other cereals

The candidate genes identified in our study and had the previous references were screened to extract out the orthologous genes in the wheat, maize and sorghum. The candidate gene stable ids were searched against the genomes, *Triticum aestivum* (IWGSC), *Zea mays* (Zm-B73- REFERENCE-NAM-5.0) and *Sorghum bicolor* (Sorghum_bicolor_NCBIv3) in “Biomart” of the Ensamble plants database to identify the orthologous genes. The gene stable id, chromosome number, gene start and end were tabulated. The GO term and its definition was extracted from the respective databases of the species for functional analysis and based on the GO terms, the genes responsible for salinity tolerance were selected for searching the previous references for salinity tolerance.

## Results

### Distribution of QTLs for salinity tolerance

A total of 768 relevant QTLs with the traits that have a significant correlation with salinity tolerance at seedling as well as reproductive stages in rice were selected to discover the associated consensus genomic regions (Supplementary file 1). The QTLs were retrieved from 34 studies having 35 independent mapping populations including RILs (12 case studies), F_2_ (10), ILs (9), F_2:3_ (2), BC (2) and landraces (1) with population sizes ranging from 61 to 281 reported from 2001 to 2021. The QTLs are identified for the 71 different trait hoods and then they are regrouped into three classes like, traits related to ionic stresses (Na^+^ and K^+^), traits associated with morphological attributes and combined analysis of salinity tolerance as a whole. The information of QTLs for all the studied traits and their distribution on the chromosomes are shown in Fig. 1A and B. Highest number of QTLs (148 QTLs) were observed on chromosome 1 followed by chromosome 2 (81 QTLs) while the lowest number of QTLs harbored by chromosome 10 (39 QTLs). Among the traits studied for salinity tolerance, salt evaluation score (SES) having the maximum number of QTLs (95 QTLs) followed by seedling dry weight (SDW, 68 QTLs) and Shoot potassium content (SK, 48 QTLs). The phenotypic variance of the QTLs studied ranged from 1.3% to 81.56% with an average of 13.88% and the LOD score ranged from 1.92 to 32 with an average of 4.43. The confidence interval for the QTLs studied was ranging from 0.3 to 89.4 cM with an average of 17.35 cM (Fig. 1C).

### The consensus map of rice

The consensus map prepared from the reference maps and genetic maps of the studies has the marker density of 57,806 with an average of 4817 markers per chromosome and the genetic map of 3041.19 cM long with an average 253.43 cM region per chromosome (Supplementary file 2). The size of the individual chromosome ranges from 153.64 cM (chromosome 11) to 408 cM (chromosome 1) and the number of markers ranges from 3220 (chromosome 10) to 7272 (chromosome 1) (Fig. 1D). The average marker density of the consensus map was 19 markers per cM with the highest on chromosome 11 (30.69 markers per cM) and the lowest on chromosome 12 (14.11 markers per cM).

### MQTLs identified for salinity tolerance traits

All of the 768 QTLs were projected on the consensus map and from it the QTLs projected outside the chromosomal region were excluded from the further analysis. Also, the QTLs lacking the appropriate marker information were excluded. The MQTL analysis summarized all of the 768 QTLs into 158 MQTLs. Moreover, MQTLs were further screened by excluding the MQTLs with less than 5 candidate QTLs to identify the true MQTLs responsible for salinity tolerance. Finally, 65 MQTLs out of 768 studied QTLs (8.43 per cent) were extracted with an average CI reduced from 17.35 to 1.66 cM (Table 3). Many of the QTLs (238 QTLs) could not be assigned to any of the MQTL due to lack of common genomic regions, in the MQTLs with less than 5 candidate QTL regions or relatively low phenotypic variance for the trait. MQTLs per chromosome ranged from 4 to 8 with an average of 5.42 MQTLs per chromosome. The highest number of MQTLs were identified on chromosomes 2 and 4 (8 MQTLs) while the lowest was on chromosome 7 (3 MQTLs). Correlation between the number of initially identified QTLs and the MQTLs density was low (0.51) suggesting MQTL identification is independent of the prior number and they are based on the CI, LOD and the phenotypic variance explained (Fig. 1B). A higher number of consensus groups does not necessarily imply greater stability of QTL. Instead, the stability of QTL is determined by various factors and should not be solely based on the number of consensus groups. The MQTL1.2 is derived from 61 different populations that have the highest number of candidate QTLs (75), followed by MQTL1.7 and MQTL1.5 (35) which may prove robust and stable in different environments and years.

**Table 3:** Summary of the MQTLs detected for salinity tolerance in rice.

MQTL name	Chr	Position (cM)	Start marker	End marker	CI (95%)	Genetic left	Genetic right	Genomic left	Genomic right	No of candidate QTLs	Candidate QTLs
MQTL1.1	1	51.58	Bin1_11.19–11.26	Bin1_11.98–12.32	0.73	51.45	51.68	11.23	12.15	28	SDW, SNA, SES, SL, RL, RNA, SFW, PH, RDW, SNAK, RK
MQTL1.2	1	102.58	Bin1_21.99–22.18	Bin1_23.5–23.74	0.1	102.40	102.69	22.09	23.62	75	SES, SH, SNAK, SK, CHL, SFD, SL, RDW, SDW, FNAK, RNA, PH, SFW, SNA, PF, RNAK
MQTL1.3	1	53.71	Dj01_13425188F	Sj01_13981650R	1.03	53.67	53.81	13.43	13.98	10	SES, RNA, SNA, SNAK
MQTL1.4	1	86.04	Sj01_21160869F	Sj01_22728151R	3.01	86.00	86.19	21.16	22.73	5	SES, SK
MQTL1.5	1	116.02	Dj01_29556725R	Dj01_29707926R	0.14	115.98	116.08	29.56	29.71	31	FNAK, SH, SNAK, SES, SK, RNA, SNA, RK, RNAK
MQTL1.6	1	53.04	Dj01_13193179F	Bin1_11.98–12.32	1.35	52.98	53.10	13.19	14.23	11	PH, RL, SFW, SDW, SES, SL
MQTL1.7	1	93.73	Dj01_23852434R	Dj01_23986664R	0.23	93.66	93.76	23.85	23.99	39	SFW, SDW, SES, SL, PH, RDW, PF, SH, CHL, RL
MQTL2.1	2	29.86	Dj02_07536262F	Dj02_07781792F	0.47	29.85	29.90	7.54	7.78	17	SES, PF, SK, SL, RDW, SNA, SDW, SFW
MQTL2.2	2	57.72	Dj02_14366103F	Bin2_10.14–10.79	0.49	57.47	58.20	14.37	15.01	18	SES, SK, SNA, RFW, SDW, RL
MQTL2.3	2	83.78	Sj02_21161357R	Dj02_21613227F	0.81	83.72	83.82	21.16	21.61	7	SL, SES, RDW, SNA, SK, SNAK, RFW
MQTL2.4	2	26.49	Dj02_06374313R	Dj02_07234989F	1.68	26.47	26.53	6.37	7.23	8	SES, SK, SNA
MQTL2.5	2	52.32	Sj02_13307122F	Dj02_13470371F	0.3	52.20	52.38	13.31	13.47	8	SFW, SES, SK
MQTL2.6	2	62.28	Sj02_15807823F	Sj02_16056120R	0.43	62.20	62.39	15.81	16.06	14	SNA, SNAK, SK, SES, RNAK
MQTL2.7	2	29.84	Sj02_07525457R	Dj02_07781792F	0.47	29.66	29.85	7.53	7.78	12	SL, SDW, PF, SES, SDS, RDW
MQTL2.8	2	83.51	Sj02_21146226F	Dj02_21461962R	0.03	83.42	83.71	21.15	21.46	14	RDW, PL, SES, IR, SL, SDW, GYLD, RL
MQTL3.1	3	30.91	Sj03_07607092R	Dj03_08259469F	1.22	30.91	30.93	7.61	8.26	7	RL, SFW, SES, RDW, BM, SK
MQTL3.2	3	57.79	Dj03_13985031R	Sj03_15864306F	4.25	57.78	57.81	13.99	15.86	9	RDW, SK, SNAK, SFW, SDW, SNA
MQTL3.3	3	86.05	Dj03_21966002R	Dj03_22049522R	0.1	86.03	86.10	21.97	22.05	16	SES, SDS, SK, SFW, SNA, SNAK, CHL, SL, SDW
MQTL3.4	3	50.68	Sj03_11108193R	Dj03_14901674R	7.43	50.67	50.69	11.11	14.90	6	RK, SES, SK, SNAK, SNA
MQTL3.5	3	20.16	Sj03_05343807R	Sj03_05349416F	0.49	20.16	20.17	5.34	5.35	5	RL, RDW, SFW, BM
MQTL3.6	3	58.53	Sj03_13841038R	Sj03_16147935F	4.55	58.41	58.53	13.84	16.15	5	RDW, SFW, SDW
MQTL3.7	3	86.29	Dj03_22049522R	Dj03_22107671R	0.08	86.28	86.39	22.05	22.11	14	PH, RL, SL, SFW, SES, SDS, SDW, CHL
MQTL4.1	4	19.47	Dj04_04170196F	Dj04_05950987R	3.54	19.44	19.50	4.17	5.95	5	SFW, GYLD, DM
MQTL4.2	4	52.5	Dj04_13251149F	Dj04_13674458R	0.72	52.44	52.60	13.25	13.67	5	SK, SNA, SNAK, RDW, SES
MQTL4.3	4	61.26	Bin4_13.97–14.12	Dj04_16001101R	1.17	60.94	61.23	14.05	16.00	8	SES, SNAK, SDW, SFW
MQTL4.4	4	80.26	Sj04_20496453R	Sj04_20593680F	0.16	80.20	80.28	20.50	20.59	13	RNA, RK, RNAK, SNAK, SDW, SL, SK, GP, IR, SFW
MQTL4.5	4	19.44	Dj04_04170196F	Dj04_05928778R	3.55	19.43	19.44	4.17	5.93	5	DM, SFW, NFS, GYLD
MQTL4.6	4	29.14	Dj04_07389255R	Dj04_07593548R	0.29	28.69	29.27	7.39	7.59	5	GP, SH, RL
MQTL4.7	4	59.66	Dj04_14237029R	Bin4_14.55–14.97	4.12	58.50	58.66	14.24	14.76	5	SES, SDW, SFW
MQTL4.8	4	80.58	Sj04_20593680F	Dj04_20683782F	0.1	80.50	80.59	20.59	20.68	5	SL, IR, SDW, SFW, GP
MQTL5.1	5	40.08	Sj05_09216782R	Sj05_11207151R	3.97	39.66	40.50	9.22	11.21	7	CHL, RL, SL, SNAK, RNAK, SNA
MQTL5.2	5	55.1	Dj05_13712994R	Sj05_14450116F	1.47	54.82	55.33	13.71	14.45	5	SDW, RDW, SES, SFW
MQTL5.3	5	82.82	Sj05_21045832R	Dj05_21302103F	0.4	82.75	82.92	21.05	21.30	15	SNAK, SFW, SES, SL, SNA, PH, BM, RDW, SK
MQTL5.4	5	54.56	Sj05_13572519F	Sj05_14344276R	1.44	54.46	54.59	13.57	14.34	8	RL, RDW, SFW, SES, SDW
MQTL5.5	5	81.68	S5_20820664	Dj05_20956305F	0.44	82.63	82.72	20.82	20.96	12	SL, SES, SFW, PF, BM, PH, RDW
MQTL6.1	6	38.97	Sj06_09382846R	Dj06_10547551F	2.24	38.86	39.03	9.38	10.55	5	SDW, SES, SL
MQTL6.2	6	70.25	Dj06_17122068R	Bin6_12.44–13.03	3.17	70.23	70.27	17.12	12.74	6	SNA, RNA, SES, RL
MQTL6.3	6	93.62	Sj06_23592921R	Sj06_24128786R	1.19	93.60	93.62	23.59	24.13	10	GYLD, SNAK, RK, RNA, SFW, RL
MQTL6.4	6	69.57	Dj06_16822772F	Dj06_18803272F	3.69	69.54	69.59	16.82	18.80	5	SNA, RNA, SES
MQTL6.5	6	39.84	Dj06_09635471F	Sj06_10727303F	2.13	39.60	39.85	9.64	10.73	8	SDW, SES, SL, SFW
MQTL6.6	6	63.1	Bin6_11.04–11.16	Sj06_16652720F	1.81	63.08	63.24	11.10	16.65	10	SDS, CHL, SES, IR, RL, SDW
MQTL6.7	6	96.2	Sj06_24039978F	Sj06_25103415R	2.05	96.09	96.20	24.04	25.10	9	GYLD, SFW, RL, RK, BM
MQTL7.1	7	61.24	Dj07_14488453R	Sj07_16873873F	4.83	61.14	61.37	14.49	16.87	9	SES, RDW, RNA, RL
MQTL7.2	7	9.47	Sj07_01801193R	Sj07_03154026R	2.49	9.42	9.55	1.80	3.15	6	RDW, SDW, SFW
MQTL7.3	7	56.92	Dj07_14488453R	Dj07_14672955R	0.38	56.89	56.96	14.49	14.67	5	SDS, SES, CHL
MQTL8.1	8	84.94	Sj08_21582839F	Dj08_21828309F	0.3	84.83	85.12	21.58	21.83	6	GYLD, RK, RNAK, RFW
MQTL8.2	8	80.14	Dj08_18717359R	Dj08_22198956R	6.79	80.01	80.17	18.72	22.20	8	SK, SNAK, SNA, SES, RK, RNAK
MQTL8.3	8	23.1	Sj08_05750246F	Sj08_06044001R	0.52	23.03	23.14	5.75	6.04	5	CHL, BM, RDW, SDW
MQTL8.4	8	84.94	Sj08_21582839F	Dj08_21747733F	0.31	84.83	85.12	21.58	21.75	6	SFW, GYLD, SL, RFW
MQTL9.1	9	24.86	Dj09_06118286F	Dj09_06831346F	1.39	24.80	24.92	6.12	6.83	9	SFW, SNA, SES, RDW, RFW, RNA, RNAK, SDW
MQTL9.2	9	41.54	Dj09_10618925R	Dj09_10752402R	0.09	41.54	41.60	10.62	10.75	7	CHL, RL, SES
MQTL9.3	9	62.92	Dj09_16057823R	Bin9_14.39–14.71	0.49	62.68	62.92	16.06	14.55	14	SES, SDS, CHL, RL, RK, RNA, RNAK, SNAK, SDW, RFW
MQTL9.4	9	61.8	Bin9_12.72–13.11	Sj09_17214079R	5.2	61.76	61.91	12.92	17.21	6	RNAK, SNAK, RK, SES, RNA
MQTL10.1	10	85.23	Dj10_21071821F	Sj10_22586330F	2.95	85.19	85.25	21.07	22.59	16	BM, CHL, SK, PF, SES, RL
MQTL10.2	10	126.18	RM8207	RM3152	0.73	126.16	126.41	9.80	10.21	6	CHL, SL, GP, IR, RL
MQTL10.3	10	84.49	Dj10_20992328F	Sj10_22266999F	2.52	84.40	84.59	20.99	22.27	13	PF, BM, CHL, RL, SES
MQTL10.4	10	126.17	RM8207	RM311	0.54	126.16	126.41	9.80	10.23	6	CHL, SL, GP, IR, RL
MQTL11.1	11	67.46	Dj11_17355923R	Dj11_17412175F	0.11	67.43	67.53	17.36	17.41	7	SNA, SL, SFW, SES
MQTL11.2	11	8.5	Dj11_02304244R	Sj11_02345272F	0.07	8.43	8.54	2.30	2.35	7	CHL, SES, SDW
MQTL11.3	11	39.01	Sj11_09688800F	Dj11_10527020F	1.65	38.99	39.18	9.69	10.53	5	SDW, RL, RDW, SES
MQTL11.4	11	67.47	Dj11_17340485F	Sj11_17434166R	0.11	67.43	67.53	17.34	17.43	6	SL, RFW, SFW, SIS, SES
MQTL12.1	12	65.97	Dj12_16350381F	S12_17681133	2.26	65.90	66.02	16.35	17.68	5	SFW, SDW, SL, IR
MQTL12.2	12	72.57	Bin12_17.05–17.16	S12_18855850	0.04	72.40	72.70	17.11	18.86	13	SH, SFW, SNA, SES, SDW, SNAK, RDW, SL, RFW
MQTL12.3	12	46.7	Bin12_9.31–9.4	Sj12_13864120F	7.15	46.23	46.78	10.39	13.86	6	SNAK, SES, SK
MQTL12.4	12	72.43	Bin12_17.05–17.16	Dj12_18705808F	0.04	72.40	72.70	17.11	18.71	10	SES, SFW, SL, SH, SDW, RDW

Highest number of MQTLs was identified for the trait salt evaluation score (SES, 46 MQTLs) with an average of 2.06 SES QTLs per MQTL. Highest number of SES QTLs found on MQTL was 1.2 (9 QTLs) while the lowest for MQTL was 2.3 (1 QTL). For the traits seedling fresh and dry weight (SDW and SFW) the MQTLs, MQTL1.1 (8 QTLs, CI: 0.73 cM), MQTL1.2 (4 QTLs, CI: 0.1 cM), MQTL1.7 (4 QTLs, CI: 0.23 cM), MQTL2.1 (4 QTLs, CI: 0.47 cM), MQTL4.3 (4 QTLs, CI: 1.17 cM) and MQTL6.5 (4 QTLs, 2.13 cM) were promising. MQTL1.7 (9 QTLs, CI: 0.23 cM), MQTL1.2 (6 QTLs, CI: 0.1 cM) and MQTL1.6 (3 QTLs, 1.35 cM) were found associated with the shoot length of the seedling under saline conditions. The root length was positively correlated with the MQTL1.6 (CI: 1.35 cM), MQTL3.1 (CI: 1.22), MQTL6.6 (CI: 1.81) and MQTL9.2 (CI: 0.09 cM). Significant MQTLs for ionic concentrations and their ratio in the shoot were MQTL1.2 (12 QTLs), MQTL1.5 (7 QTLs), MQTL2.6 (6 QTLs) and MQTL1.1 (4 QTLs). While for ionic concentrations in the root, MQTL1.2 (6 QTLs), MQTL1.5 (6 QTLs), MQTL4.4 (4 QTLs) and MQTL9.4 (4 QTLs) were responsible with the CI of 0.1 cM, 0.14 cM, 0.16 cM and 5.2 cM, respectively (Fig. 1D). As the tolerance to salinity of the plants against salt stress involves complex interconnecting signaling pathways that ultimately lead to the development of measurable phenotypes. The seedling and reproductive stage salinity traits are the results of these pathways. The MQTLs explained further are based on the mechanisms of the salinity tolerance for which we have identified consensus genomic regions and the candidate genes that ultimately complete the network (Fig. 2).

### Identification of candidate genes for MQTLs

Exploring all the 64 MQTL regions for candidate genes resulted in the identification of 6973 candidate genes spanning all the chromosomes (Supplementary file 3). The highest number of candidate genes were identified on chromosome 6 (689 genes) while the lowest was on chromosome 11 (365 genes). The enrichment of the identified candidate genes enabled to filter them into three classes *i.e.* biological processes, molecular function and cellular components. Among them, 553 candidate genes were identified based on their potential role in salinity tolerance in rice. After screening the genes from the previous references, 56 candidate genes were identified which express in shoot, root and reproductive parts imparting salinity tolerance at seedling and reproductive stages (Table 4). While, the remaining 497 unique genes may have a potential role in maintaining osmotic and ionic balance during salinity stress.

**Table 4:** Summary of candidate genes identified from MQTLs for salinity tolerance in rice.

MQTL	Gene stable ID	Symbol	Chr	Function	References
MQTL1.1	Os01g0303600	OsRFP	1	During plant responses to environmental stress, it helps in the post-translational alteration of target proteins	Lim et al. 2013^15^
MQTL1.1	Os01g0304100	OsCCC2	1	The prevalence of cation-chloride cotransporters and their significance in major developmental processes and Cl- homeostasis in plants	Flores et al. 2007^49^
MQTL1.1	Os01g0307500	OsHKT1;5, SKC1, OsHKT8, OsHK1;5	1	Salinity tolerance is determined by the HKT1;5 loci/alleles. HKT1;5 s are plasmalemma-localized Na + transporters that transfer xylem Na + into xylem parenchyma cells, lowering shoot Na + buildup	Somasundaram et al. 2020^44^
MQTL1.1	Os01g0309800	OsHypB	1	ABA, ethylene, jasmonic acid, salt and drought stress have all been proven to increase H2 generation	Zeng et al. 2013^41^
MQTL1.2	Os01g0582400	OsCYP	1	OsCyp2-P is a promising candidate gene for improving various abiotic stress tolerance as it works by scavenging reactive oxygen species (ROS) and maintaining ion homeostasis	Kumari et al. 2015^89^
MQTL1.4	Os01g0566100	OsEF3, OsELF3-2, OsELF3.2	1	Important function in root development, grain weight and days to heading	Wang et al. 2021a^90^
MQTL1.4	Os01g0570800	OsIQM	1	PEG, NaCl, jasmonic acid (JA) and abscisic acid (ABA) administration elicited responses in most IQM genes, implying that they play important roles in biotic and abiotic stress responses	Fan et al. 2021^91^
MQTL1.4	Os01g0571300	OsHsfA7	1	Over-expressing OsHsfA7 rice demonstrated reduced damage symptoms and higher survival rates, leaf electrical conductivity and malondialdehyde levels when exposed to salt	Liu et al. 2013^92^
MQTL1.5	Os01g0711600	OsRTH1	1	OsRTH1 modifies ethylene responses, revealing the biological importance of ethylene in rice seedling growth and development	Zhang et al. 2012^62^
MQTL1.6	Os01g0350900	OsIPI1	1	Plant architecture was significantly altered in the ipi1 loss-of-function mutants, with more tillers, enlarged panicles and higher yield per plant	Wang et al. 2017^93^
MQTL1.6	Os01g0353400	OsGST4	1	The ROS-scavenging activity of OsGST4 protein was identified and its mutant exhibited delayed growth and a high vulnerability to salt and oxidative stress	Xu et al. 2018^34^
MQTL2.3	Os02g0558100	OsCLC-2	2	OsCLC-1 aids avoidance of chloride ions by accumulating them in vacuoles	Nakamura et al. 2006^50^
MQTL3.1	Os03g0244600	OSLAX	3	OsRAU1 is involved in the increased phloem auxin translocation in lateral roots and their primordia, which speeds up lateral root development	Chhun et al. 2007^94^
MQTL3.2	Os03g0366200	OsCBK	3	OsCBK is prevalent in sporogenous cells of the anther during meiosis and is significantly expressed in cell division zones	Li et al. 2006^95^
MQTL3.2	Os03g0367000	OsCYP	3	OsCYPs were shown to up-regulate a large number of genes in response to salt and desiccation stress	Ahn et al. 2010^31^
MQTL3.3	Os03g0593200	CBSDUF	3	CDCPs are important in stress response/tolerance as well as development	Kushwaha et al. 2009^96^
MQTL3.4	Os03g0318600	OsbZIP28, OsbZIP1	3	In response to salicylic acid, jasmonic acid and abscisic acid, OsbZIP1 is constitutively produced in the roots and highly stimulated in rice leaves	Hasegawa et al. 2021^37^
MQTL3.4	Os03g0319300	OsCam1-1	3	Calmodulin has been found to have a part in the signal transduction cascade in proline accumulation during salt stress and ABA has been shown to upregulate OsCam1-1 (the salt-stress-responsive calmodulin) gene expression	Yuenyong et al. 2018^40^
MQTL3.4	Os03g0320600	OSVQ	3	In plants treated with abscisic acid, OsVQ genes operate as essential co-regulators during the plant defense response to biotic and abiotic stresses	Kim et al. 2013^39^
MQTL3.4	Os03g0321700	OsWRKY55	3	OsWRKY55 was found to be expressed in osmotic and abscisic acid (ABA) treatments and to play a vital role in rice plant height regulation	Huang et al. 2021^38^
MQTL3.4	Os03g0332400	OsGLYII2	3	Increased photosynthesis and lower oxidative damage under stress circumstances appear to be the mechanism allowing for increased stress tolerance	Ghosh et al. 2014^97^
MQTL3.4	Os03g0337500	OsHAK8	3	OsHAK8, a rice potassium transporter, is involved in K + uptake and translocation. Widely expressed in roots and the protein was directed to the plasma membrane	Wang et al. 2021b^98^
MQTL4.1	Os04g0169100	Os-ERL1, OsETR2	4	Abiotic stresses and phytohormones govern OsARD expression, which is expressed in roots under flood circumstances and inhibited by abiotic stresses such as water deficit, excessive salt and low temperature	Lin et al. 2005^63^
MQTL4.1	Os04g0176400	OsSCP	4	Several pollen-specific elements were discovered during the search for promoter regions and these promoters were active in mature pollen grains and pollen tubes. OsSCPs play a key part in the maturation of mature pollen and the creation of pollen tubes	Park et al. 2006^51^
MQTL4.2	Os04g0304400	OsMADS25	4	In the presence of nitrate, MADS-box Transcription Factors greatly increases primary root length, lateral root number, lateral root length and shoot fresh weight	Xu et al. 2011^99^
MQTL5.2	Os05g0312600	CML	5	Increased tolerance to excessive salt and drought was associated with altered expression of stress/ABA-responsive genes when OsMSR2 was expressed	Ahmadi et al. 2011^46^
MQTL6.2	Os06g0504100	MADS	6	In rice, MADS-box genes are expressed during reproductive development and stress	Ahmadi et al. 2011^46^
MQTL6.2	Os06g0504900	OsWRKY	6	The OsWRKY31 gene was discovered to promote lateral root development and elongation when it was overexpressed	Zhang et al. 2008^100^
MQTL6.3	Os06g0603000	PE-1	6	PE-1 was found mostly in roots, stems, leaves, leaf sheaths and juvenile panicles and was associated with lower chlorophyll concentration, increased photosynthesis and lower pollen fertility	Rao et al. 2019^54^
MQTL6.3	Os06g0603600	OsSPX1	6	Rice seedlings with high OsSPX1 levels are resistant to cold and oxidative stress. Rice normal anther and pollen development was hampered by OsSPX1 downregulation, which disrupted glucose metabolism and sugar transport, resulting in semi-male sterility and reduced seed-setting rate and grain yield	Zhang et al. 2016^35^
MQTL6.3	Os06g0606000	OsSOS2	6	SOS2 overexpressing plants were found to have better ion and redox homeostasis in the presence of salinity and it plays a function at both the seedling and reproductive stages	Kumar et al. 2022^45^
MQTL6.4	Os06g0506600	OsUBC	6	Drought, salt stress and ABA dramatically up-regulated UBC genes involved in hormone-mediated stress responses, which were preferentially expressed in leaves, panicles and/or seeds	EZ et al. 2015^101^
MQTL6.6	Os06g0335500	IAA	6	Many genes were responsive to diverse abiotic stimuli, demonstrating that plant development and abiotic stress interact, as evidenced by the root growth of transgenic rice	Song et al. 2009^102^
MQTL6.6	Os06g0348800	OsGLK1	6	OsGLK1 is a crucial regulator of chloroplast development, since it governs chloroplast development under the control of light and phytohormones	Nakamura et al. 2009^103^
MQTL6.7	Os06g0610350	MOC1	6	The MONOCULM 1 (MOC1) gene was discovered to be the first important regulator of rice tiller number	Lin et al. 2012^53^
MQTL7.1	Os07g0448100	OsPIP2	7	OsPIP2;2 performed a role in cell membrane integrity and efficiently protected rice cells from osmotic stress-induced electrolyte leakage	Bai et al. 2021^104^
MQTL7.1	Os07g0451300	OsNAC45	7	OsNAC45 played an important role during ABA signal responses by reducing ROS accumulation in roots and increased salinity tolerance in rice	Zhang et al. 2020a^105^
MQTL7.2	Os07g0150100	OsDDP	7	Under salinity stress, OsDDPs were differently regulated, with OsDDP6 being increased at all developmental stages in the salt tolerant rice genotype FL478	Ganie et al. 2017^59^
MQTL8.1	Os08g0445700	OsTPS	8	Through ABA signaling, OsTPS8 may modulate suberin deposition in rice and Salinity tolerance is also aided by SAPK9-mediated regulation of altered ABA-responsive genes	Vishal et al. 2019^106^
MQTL8.2	Os08g0409100	OsTPP	8	Drought, salt and cold resistance have been demonstrated in trehalose producing genes	Iordachescu and Imani, 2008^107^
MQTL9.1	Os09g0286400	OsNHX	9	In lateral roots, the vascular bundle, the water pore and the basal section of seedling shoots, OsNHX1 or OsNHX5 promoter activity was seen. Salt stress, hyperosmotic stress and ABA increase the expression of OsNHX1, OsNHX2, OsNHX3 and OsNHX5 in rice tissues in distinct ways	Fukuda et al. 2011^48^
MQTL9.3	Os09g0439200	OsJAZ8, OsTIFY10c	9	OsJAZ8 is associated with enhanced salt tolerance, demonstrating the importance of jasmonate signaling during stress tolerance	Peethambaran et al. 2018^108^
MQTL9.3	Os09g0448200	OsHAK	9	McHAK1 and McHAK4 have a role in maintaining potassium levels in leaves and roots during salt stress and their expression is enhanced in leaves and roots in response to excessive salinity	Ahmadi et al. 2011^46^
MQTL9.3	Os09g0455200	OsHsf	9	By increasing ABA sensitivity and temporal modulation of salt responsive genes involved in signaling and ion homeostasis, OsHsfC1b improves salt and osmotic stress tolerance	Schmidt et al. 2012^109^
MQTL9.3	Os09g0457100	OsABA	9	In rice, the OsABA8ox3 gene is critical for modulating ABA levels and osmotic stress tolerance	Cai et al. 2015^36^
MQTL9.4	Os09g0393200	OsJMJ	9	Under salt treatment, JMJ-C members were highly expressed in the flag leaf stage of FL478	Chowrasia et al. 2018^52^
MQTL9.4	Os09g0416200	OsGMST	9	Under salt stress, OsGMST1 was upregulated and knocking it out in rice resulted in hypersensitivity to salt stress	Deng et al. 2019^110^
MQTL10.1	Os10g0544900	OsPP2Cs	10	The majority of PP2C genes are involved in stress tolerance, particularly the ABA response	Xue et al. 2008^111^
MQTL10.1	Os10g0553300	OsTPS1	10	Rice seedlings with higher trehalose and proline concentrations when OsTPS1 was overexpressed showed more resistance to cold, high salinity and drought	Li et al. 2011^112^
MQTL10.1	Os10g0560400	OsCCT	10	CCT family genes regulated the heading date under both long day and short-day conditions	Zhang et al. 2015^113^
MQTL10.1	Os10g0563600	OsMSRA	10	The key involvement of OsMSRA4.1 is in the fight against oxidative stress and salt tolerance	Guo et al. 2009^33^
MQTL10.1	Os10g0564800	OsCBL1	10	OsCBL1 regulates rice seedling growth and regulates lateral root elongation by modulating auxin production	Yang et al. 2019^114^
MQTL11.3	Os11g0284900	OsWRKY	11	WRKY genes play a role in regulating ABA responses in plants	Xie et al. 2005^115^
MQTL11.3	Os11g0490900	OsApx	11	Abiotic stress, such as salt, heat, strong light and methyl viologe, induces APXs (APx1/2 s) to change redox homeostasis (increased levels of H2O2 and ascorbate)	Bonifacio et al. 2011^32^
MQTL12.1	Os12g0478200	OsABA	12	Drought and salt were found to induce the expression of the OsABF1 gene, which encodes a bZIP transcription factor, in seedling shoots and roots	Hossain et al. 2010^9^
MQTL12.2	Os12g0489100	OsMB	12	OsM4 and OsMB11 are substantially expressed in drought and salinity stress, they could be used to develop stress-resistant crops	Kushwaha et al. 2016^96^

### MQTLs and candidate genes for ROS scavenging

Accumulation of reactive oxygen species (ROS) is responsible for the cellular death and drying of the vegetative parts of the plants. MQTL1.2, MQTL1.6, MQTL6.3, MQTL10.1 and MQTL11.3 were identified to be responsible for the fight against oxidative stress and redox homeostasis with the candidate QTLs 75, 11, 10, 16 and 5, respectively. The genes underlying these QTLs, OsCYP, OsGST4, OsSPX1, OsMSRA and OsApx found to be involved in ROS-scavenging. The traits associated with the ROS detrimental effects are salt evaluation score (SES), chlorophyll content (CHL), root dry weight (RTDW), pollen fertility (PF), total spikelets (TSP) and spikelet sterility (STE)^31–35^.

### MQTLs and candidate genes for osmotic stresses

At seedling and reproductive stages, the salinity stress occurs as osmotic and ionic stress. MQTL1.1, MQTL3.1, MQTL3.4, MQTL6.4, MQTL7.1, MQTL9.3 and MQTL10.1 were found responsible for the accumulation of osmolytes and stress-responsive hormones such as ABA, jasmonic acid, salicylic acid, trehalose, PEG and proline, which govern traits such as salt evaluation score (SES), survival days to seedling (SDS), Relative shoot dry weight (RLSDW), shoot fresh/dry weight (SFD), Imbibition rate (IR), shoot fresh weight (SFW), shoot K^+^ content (SK) and root K^+^ content (RK). The initial QTLs under that MQTLs ranges from 5 to 28 including the important candidate genes responsible for osmotic stresses. The gene OsABA on chromosome 9 (MQTL9.3) was found to be critical in modulating the ABA levels during the osmotic stresses^36^. As the increase in ABA level, triggers the signaling pathways that upregulate many of the stress-responsive genes viz., OsHybP (MQTL1.1), OsZIP (MQTL3.4), OsCam (MQTL3.4), OsVQ (MQTL3.4), OsWRKY55(MQTL3.4), OsCML (MQTL5.2), etc. that are actively involved in osmotic stress tolerance^37–41^. The MQTL1.5 on chromosome 1 harbouring the gene OsRTH is responsible for ethylene responses, revealing its importance in seedling growth and development during stress conditions^42^.

### MQTLs and candidate genes for ionic stresses

The saltol is the most reviewed major QTL with 43.7% phenotypic variance for Na^+^/K^+^ homeostasis and is responsible for the salinity tolerance at the seedling stage^43^. This region is identified from the landrace Pokkali showing high tolerance to salinity and also a similar region was identified from the landrace Nona Bokara. In this work, MQTL1.1 on chromosome 1 between 11.23 Mb and 12.15 Mb was discovered which is in the same region as saltol and SKC, with a modest confidence interval of 0.92 Mb and 28 candidate QTLs. Furthermore, compared to the initial identification of that locus, this found area has lowered the confidence interval from 32.7 to 0.73 cM. In previous investigations, the candidate genes Os01g0303600 (OsRFP), Os01g0304100 (OsCCC2), Os01g0307500 (OsHKT1;5, SKC1, OsHKT8) and Os01g0309800 (OsHypB) from this area validated their significance in salinity tolerance. Because the OsHKT and OsSKC genes are responsible for Na^+^ transporters located in the plasmalemma, they move sodium from the xylem to the parenchyma cells, limiting Na^+^ buildup in the shoot^44^.

SOS2, a major component of the SOS pathway is required to maintain intracellular Na^+^ and K^+^ homeostasis during the salinity stress and is found in MQTL6.3 on chromosome 6. SOS2 was found to be working for both seedling and reproductive stage salinity tolerance^45^. A potassium transporter, OsHAK present on MQTL3.4 and MQTL9.3 is involved in K^+^ uptake and translocation involved in ion homeostasis^46^. Under salinity stress, OsHAK21 is required to maintain Na^+^/K^+^ homeostasis and support seed germination and seedling establishment^47^. The NHX genes that are Na^+^/H^+^ antiporters associated with tissue tolerance are found in MQTL 9.3 with 14 candidate QTLs^48^. Many of the chloride transporters were found to be active as a response to the salinity stress. Among them, OsCCC2 (MQTL1.1) has significance in developmental processes and Cl^-^ ion homeostasis^49^. Higher accumulation of Cl^-^ ions in the cytosol is limited by sCLC-2 (MQTL2.3) by accumulating them into vacuoles^50^.

### MQTLs and candidate genes for reproductive stage salinity traits

At reproductive stage salinity tolerance, the major effects of salt are observed in pollen development and maturity which reduces the number of fertile spikelets and ultimately the grain yield. The MQTL4.1, MQTL6.3 and MQTL10.2 on chromosomes 4, 6 and 10 were deduced from 5, 10 and 6 initial QTLs, respectively. They collectively harbored 27 screened genes including OsSCP and OsSPX1 among which the OsSCP is responsible for pollen maturation and creation of pollen tube while, OsSPX is responsible for glucose metabolism and sugar transport during the pollen development^35,51^. The MQTL9.4 on chromosome 9 had the members of gene OsJMJ-C having higher expression in flag leaf during salinity stress^52^. The MQTL6.7 was found to be linked with the grain yield per plant under salt stress conditions including nine initial QTLs having the gene MOC1 which is one of the important regulators of tiller number^53^. MQTL6.3 with 10 candidate QTLs was found to be responsible for panicle number, 1000 grain weight, biomass content and grain yield per plant. OsWRKY, PE-1 and OsSOS2 are candidate genes (MQTL6.3) discovered in roots, stems, leaves and leaf sheaths that have been linked to lateral root development, elongation, improved photosynthesis and reproductive stage salinity tolerance^35,54^.

### Orthologous genes identified in other cereals

After exploring the Wheat, Maize and Sorghum databases, 48, 23 and 47 orthologues were identified, respectively. Among them, only six genes were having previous references against salinity tolerance and the others are new potential genes that may have a role in salinity tolerance. Wheat gene TraesCS7D02G374400 (chromosome 7D), an orthologue of Os06g0606000 (Rice chromosome 6) is responsible for sensing and signalling to osmotic stress (Yue et al. 2021)^55^. Zm00001eb197200_T003 (chromosome 4), a rice orthologue (Os01g0304100) of maize has a role in maintaining osmotic balance during salinity stress. While in sorghum, an orthologue SORBI_3001G389700 on chromosome 1 (Os03g0320600) was having a role in response to osmotic stress. For potassium ion transmembrane transporter activity, orthologous gene in maize, Zm00001eb016160 on chromosome 1 (Os03g0337500) was found responsible for the transfer of potassium ions (K^+^) from one side of a membrane to the other. While in wheat and sorghum, the genes TraesCS4A02G136300 (chromosome 4A, Os03g0337500) and SORBI_3001G379900 (chromosome 1, Os03g0337500) were found to be associated with potassium ion transport^56^. The gene MOC1 for active tillering responsible for active meristem initiation and secondary shoot formation was found in wheat and rice but not in maize and sorghum^57^. The list of orthologous genes, its GO term and GO definition is given in supplementary files 4, 5 and 6.

## Discussion

The MQTL analysis identifies the true QTLs from the initial QTL studies that had unevenly distributed and varying genetic regions associated with salinity tolerance. Detection of the MQTLs was found to be independent of the genetic background and with very low environmental effects, as they are identified from a large number of different mapping populations (35 populations) developed from the paired crossing of 51 different genotypes. These populations were taken from the studies (2001–2022) covering 22 years of independent phenotyping, reducing its environmental effects in MQTL analysis. Along with the phenotypic screening, the marker density of the genetic map is also a limiting factor to identify the true QTLs with minimum confidence interval (CI). The consensus map developed in this study is much more informative as it includes the genetic maps from numerous studies with diverse molecular markers including SNPs with the marker density as high as 56,897 (GBS study)^14,26^. As a result, MQTLs were identified with the average confidence interval reduced from 17.35 to 1.66 cM including the smallest of 0.01 cM. To identify MQTLs, association mapping study was also included and compared with the derived MQTLs, which helped to locate the true physical regions (Mb) from the genetic ones (cM). Therefore, the MQTL analysis is the best reliable method to identify the true genomic regions underlying salinity tolerance and can be used very efficiently in marker-assisted backcrossing (MABC).

MQTL analysis reduced initial 768 QTLs into 65 MQTLs present on all of the twelve chromosomes, indicating the power of MQTL analysis in narrowing down the genomic regions controlling salinity tolerance. The MQTL1.2 harboured as high as 75 candidate QTLs suggesting the robustness of the method to identify consensus regions. Here, 33 out of 65 MQTLs (CI < 1 cM) were identified with minimum linkage drag qualified as breeders QTLs, have potential use in MABC programmes. The MQTLs analysis ran separately for morphological traits, traits related to ionic concentrations and all the traits combined to ensure that, all the major regions responsible for salinity tolerance were captured. However, assuming that a lot of traits investigated are pleiotropically associated, it is more effective and robust to pool diverse correlated traits reported in the same population^21^. The analysis and interpretation of the target traits as a whole were more potent than analyzing them individually, which allows to identify the hotspots for salinity tolerance for osmotic and ionic stress that gives a large number of phenotypic effects on different traits^14^. In order to transfer the salinity tolerance, one must have knowledge about the region responsible for osmotic stress, ionic stress or a combination of both. Our approach to identify the MQTLs for individual traits and then classifying the target traits into correlated morphological, physiological and biochemical aspects, resulted in more efficient interpretation of the salinity tolerance, identifying the candidate genes and to understand the salinity tolerance mechanism as a whole.

Mechanism of osmotic tolerance is induced by the signals which ultimately leads to reduced root and shoot growth before Na^+^ accumulation in plant parts. These signals are responsible for the drought aspect of the salinity tolerance and maintenance of osmotic balance during stress conditions^58^. In this study, we have identified MQTLs harboring the candidate gene (OsABA, MQTL9.3) responsible for ABA-dependent osmotic stress signaling, causes a surge in ABA levels during salt stress. The identified genes responsible for the ABA-dependent pathway of the stress signaling are OsHybP (MQTL1.1), OsZIP (MQTL3.4), OsVQ (MQTL3.4), OsWRKY55(MQTL3.4), OsCML (MQTL5.2), etc., whose levels of expression changes due to ABA levels. Surge in the levels of ROS and accumulation of osmoprotectants are responsible for signaling in ABA-independent pathways, includes the genes OsCYP (MQTL1.2), OsGST4 (MQTL1.6), OsGLYII2 (MQTL3.4), OsSPX (MQTL6.3), OsMSRA (MQTL10.1), OsAPX (MQTL12.1) responsible for ROS producing/scavenging enzymes, some TFs, kinases etc. Apart from these, accumulation of the diverse signaling molecules such as calcium ions, hormones and osmoprotectants maintains osmotic adjustment, homeostasis and regulates plant growth and development under salinity stress for which, the identified genes are OsSOS2 (Ca2 + spike, MQTL6.3), OsCam (ABA spike, MQTL3.4) OsJAZ (Jasnmonate spike, MQTL9.3) and OsTIFY10c (Jasmonate signaling, MQTL9.3).

The identification of various sodium, potassium and chloride transporters for ionic stress tolerance are of great importance as they maintain the Na^+^ and Cl content below the damaging level in the cytosol. The identified MQTLs explicitly described the regions responsible for ion homeostasis mechanisms. The MQTL1.1 region (11.23–12.15 Mb) responsible for Na^+^/K^+^ homeostasis was identified as a comparable region to saltol and was narrowed down from 10.3–15.3 Mb^24,59^. The important candidate genes OsHKT1,5 (K^+^ transporter), SKC1 (K^+^ transporter) and OsHKT8 (Na^+^/K^+^ symporter) were all present in the MQTLs, demonstrating that the essential regions were not lost even when the confidence interval was reduced. The Na^+^/K^+^ symporter or Na^+^ uniporter (OsHKT, MQTL1.1) is present in the plasma membrane and expressed in xylem parenchyma limiting the Na^+^ accumulation in photosynthetic parts of the plant. The identified Na^+^/H antiporter OsNHX (MQTL9.1) enhances the salt tolerance by compartmentalizing the Na^+^ into the vocules^60^. High-affinity K^+^ transporter (OsHKT, MQTL1.1) and the high-affinity K^+^ uptake (OsHAK, MQTL9.3) mediate the cytosolic spike in potassium ions to regulate the homeostasis in plants under salt stress. OsHAK is also to be responsible for the accumulation of Na^+^ in old leaves. OsCLC (Cl– channel) present on vocule, responsible for Cl sequestration to vocules and expressed only in the roots, nodes, internodes and leaf sheaths were identified in MQTL2.3^50^. These are the genes and MQTLs significantly associated with the salinity tolerance and have the potential to use in marker-assisted selection to improve tolerance to salinity. For the reproductive stage salinity tolerance, highly correlated traits for salinity tolerance are pollen viability and stigma receptivity to increased Na^+^ concentration in floral parts^61^. We identified the important genes for pollen and anther development in MQTL4.1(OsSCP) and MQTL6.3 (OsSPX). The MQTL area discovered (MQTL6.3) for grain yield per plant with a CI of 0.54 Mb in the study shows a comparable region from the previous study^30^.

The rice orthologous gene of OsSPX in wheat (TraesCS7D02G374400) located on chromosome 7D is responsible for maintaining osmotic balance under salinity conditions also identified in comparative transcriptomics study^55^. The identified orthologous for OsHAK in the wheat (TraesCS4A02G136300) chromosome 4A, maize (Zm00001eb016160) chromosome 1 and sorghum (SORBI_3001G379900) chromosome 1 were also reported previously as an important potassium transporter of the plasma membrane^56,62^. These potential candidate genes have a crucial role in tolerance to salinity as they have been found in four of the major cereal crops of the Poaceae family and potential to use in MAS to improve the genotypes for salinity tolerance.

To the best of our knowledge, this is the first comprehensive study to report the MQTLs with very small confidence intervals including the major candidate genes identified for the salinity tolerance and also being previously reported. These results have a huge possibility to improve the rice genotypes for salinity tolerance.

## Conclusion

In the present investigation, a total of 65 MQTLs were extracted with an average CI reduced from 17.35 to 1.66 cM including the smallest of 0.01 cM. Identification of the MQTLs for individual traits and then classifying the target traits into correlated morphological, physiological and biochemical aspects, resulted in more efficient interpretation of the salinity tolerance, identifying the candidate genes and to understand the salinity tolerance mechanism as a whole. The results of this study have a huge potential to improve the rice genotypes for salinity tolerance with the help of MAS and MABC.

### Supplementary Information

Supplementary Information 1.

Supplementary Information 2.

Supplementary Information 3.

Supplementary Information 4.

Supplementary Information 5.

Supplementary Information 6.

Supplementary Information 7.

## Data Availability

The data that support the findings of this study are openly available. The authors confirm that the data supporting the findings of this study are available within the article [and/or] its supplementary materials.
