# Author Correction: Meta-analysis of identified genomic regions and candidate genes underlying salinity tolerance in rice (*Oryza sativa* L.)

**DOI:** 10.1038/s41598-024-58211-7

**Published:** 2024-04-09

**Authors:** Pratik Satasiya, Sanyam Patel, Ritesh Patel, Om Prakash Raigar, Kaushal Modha, Vipul Parekh, Haimil Joshi, Vipul Patel, Ankit Chaudhary, Deepak Sharma, Maulik Prajapati

**Affiliations:** 1 Department of Genetics and Plant Breeding, N. M. College of Agriculture, Navsari Agricultural University, Navsari, Gujarat India; 2 School of Agricultural Biotechnology, Punjab Agricultural University, Ludhiana, Punjab India; 3 Kishorbhai Institute of Agriculture Sciences and Research Centre, Uka Tarsadia University, Bardoli, Gujarat India; 4 Department of Biotechnology, College of Forestry, Navsari Agricultural University, Navsari, Gujarat India; 5 Coastal Soil Salinity Research Station Danti-Umbharat, Navsari Agricultural University, Navsari, Gujarat India; 6 Regional Rice Research Station, Vyara, Navsari Agricultural University, Navsari, Gujarat India

Correction to: *Scientific Reports* 10.1038/s41598-024-54764-9, published online 08 March 2024

The original version of this Article contained an error in the name of the author Om Prakash Raigar which was incorrectly given as Om Prakash. The original Article has been corrected.

