# Supplementary material for: Meta-analysis of identified genomic regions and candidate genes underlying salinity tolerance in rice (Oryza sativa L.)
Source: Sci Rep. 2024 Mar 8;14:5730. doi: 10.1038/s41598-024-54764-9 (PMC10923909; doi:10.1038/s41598-024-54764-9)
Supplement: Supplementary file 7 — Supplementary Information 7. [file 41598_2024_54764_MOESM7_ESM.docx]

**Supplementary Material**

**Supplementary files legends**

**Supplementary file 1** Candidate and MQTLs details

**Supplementary file 2** Consensus map

**Supplementary file 3** Gene ontology

**Supplementary file 4** Orthologues in wheat

**Supplementary file 5** Orthologues in maize

**Supplementary file 6** Orthologues in sorghum
